# Phenotypic Definition Influences Genetic Inference and Predictive Ability for Reining Performance in Quarter Horses Using Random Regression Models

**DOI:** 10.1111/asj.70223

**Published:** 2026-07-23

**Authors:** Mário Luiz Santana, Annaiza Braga Bignardi

**Affiliations:** ^1^ Grupo de Melhoramento Animal de Mato Grosso (GMAT), Instituto de Ciências Agrárias e Tecnológicas Universidade Federal de Rondonópolis (UFR), Av. dos Estudantes, 5055, Cidade Universitária Rondonópolis Mato Grosso Brazil

**Keywords:** genetic parameters, heritability, predictive validation, rank, transformation

## Abstract

Reining performance is influenced by subjectivity and competition structure, challenging genetic evaluation. This study estimated genetic parameters for reining performance in Brazilian Quarter Horses using random regression models (RRM) and evaluated how alternative phenotypic definitions affect genetic inference and predictive ability. A dataset comprising 21,165 longitudinal records from 1603 horses was analyzed, comparing within‐group rank (RANK) and Blom‐transformed rank (BLOM). Model comparison indicated that cubic RRM provided the best fit for both phenotypes. Heritability estimates were low and age‐dependent, ranging from 0.01 to 0.17 for RANK and from 0.06 to 0.23 for BLOM. Permanent environmental and rider effects accounted for substantial phenotypic variance. Genetic correlations were higher between adjacent ages and declined across distant ages. RANK showed greater additive genetic stability across ages and slightly higher theoretical accuracy for total EBV among selected stallions. In contrast, BLOM showed greater consistency for the combined additive genetic + permanent environmental component, stronger favorable genetic trends, and better LR‐based predictive performance across several age classes. Overall, both rank‐based phenotypes were useful and complementary for genetic evaluation of reining performance. BLOM showed modest advantages for several criteria, whereas RANK retained relevant information for representing relative competitive performance on the observed scale.

## 1 Introduction

Reining is a technically demanding equestrian discipline in which horses are evaluated on their ability to execute a predefined pattern of maneuvers with precision, fluidity, and responsiveness under saddle. Performances are judged based on the quality of movements such as spins, sliding stops, rollbacks, and lead changes, as well as on overall attitude and ease of control. Unlike speed‐based events, reining emphasizes finesse, coordination, and athletic control (Landsbergen et al. 2024; Pfau et al. 2022), positioning it among the most structured and standardized disciplines in western riding. Its global expansion, supported by organizations such as the National Reining Horse Association (NRHA, nrha.com/), Reining Australia (reiningaustralia.com.au/), and the *Associação Nacional do Cavalo de Rédeas* (ANCR, ancr.org.br/), has contributed to increased economic relevance and competitive intensity, particularly in countries with well‐established Quarter Horse populations, such as the United States, Australia, and Brazil.

Despite the global expansion and increasing competitive relevance of reining, quantitative genetic studies specifically focused on this discipline remain scarce. Most advances in equine genetics have concentrated on racing, show jumping, or general sport horse evaluations (Berglund et al. 2024; Chapard et al. 2025; Doyle et al. 2022; Velie et al. 2016), leaving western performance disciplines comparatively underexplored (Santana et al. 2025). Previous genomic studies have shown that the Quarter Horse has undergone genetic differentiation across performance disciplines, reflecting long‐term directional selection and discipline‐specific breeding goals (Beltrán et al. 2015; Meira et al. 2013; Petersen et al. 2014). In this context, Petersen et al. (2014) showed that reining horses are part of a differentiated stock‐type cluster within the Quarter Horse breed, together with cutting and working cow horses, whereas Avila et al. (2018) identified signatures of selection in the reining subpopulation and candidate genes potentially related to athletic coordination, neuromuscular function, and performance specialization. However, these studies addressed population structure and genomic selection signatures rather than the evaluation of alternative phenotypes and appropriate models for formal genetic evaluation of reining performance. This gap is particularly relevant because reining is a multifactorial judged trait influenced by precision, motor control, neuromuscular coordination, metabolic efficiency, rider–horse interaction, and competition‐specific environmental conditions (Beltrán et al. 2015; Meira et al. 2014). Therefore, statistical models capable of accounting for genetic variability, environmental influences, and the specific structure of reining competition records are needed.

A major challenge in the genetic evaluation of equestrian performance lies in the nature of the phenotypic records (Chapard et al. 2023; Ricard and Legarra 2010). Scores assigned by judges are inherently subjective, often bounded by a predefined scale, and influenced by the competitive context in which animals are evaluated. As a result, such data frequently exhibit departures from normality, heterogeneity of variance, and scale dependence across events. These issues are widely recognized in equine breeding studies and have motivated the use of alternative phenotypic definitions, including rank‐based measures and normal score transformations, such as the Blom transformation, to improve statistical properties and model assumptions (Cervantes et al. 2020; Mezei et al. 2015; Novotná et al. 2015). However, these transformations may alter the underlying genetic structure of the trait (Novotná et al. 2015; Posta et al. 2009). In this context, random regression models (RRM) provide a flexible and robust alternative for the genetic evaluation of performance traits measured repeatedly over time or across stages of development. This approach has been successfully applied in equine studies, especially when performance evolves dynamically and cannot be adequately represented by a single measurement (Gómez et al. 2011; Posta et al. 2009, 2010). For reining horses, whose performance may change with age, training intensity, and competitive experience, RRM offer a biologically and statistically coherent strategy for capturing this longitudinal variation. Thus, the present study aimed to (i) estimate genetic parameters for reining performance in Brazilian Quarter Horses using RRM and (ii) evaluate the impact of alternative phenotypic definitions on genetic inference and the predictive ability of estimated breeding values. This approach allows for a comprehensive assessment of how phenotypic scaling influences variance components, genetic relationships across ages, and the predictive performance of genetic evaluations, thereby contributing to the development of more reliable selection strategies for this discipline.

## 2 Materials and Methods

This study was based exclusively on a pre‐existing competition database, with no direct handling, experimental intervention, or biological sampling of animals by the authors; therefore, approval by an animal ethics committee was not required.

### 2.1 Data

The original dataset was provided by the *Associação Nacional do Cavalo de Rédeas* (ANCR, Holambra—SP, Brazil) and initially comprised 30,758 final score records from 2421 Quarter Horses that competed in 228 reining events held in Brazil between 2007 and 2025. In these events, horses compete individually within classes defined by specific event and category conditions, performing one of the 18 standardized reining patterns officially established by the NRHA and selected for each competition class (ANCR/NRHA, https://nrha.com/handbook/). An example of three official ANCR/NRHA patterns is presented in Supporting Information S1. In this population, selection has historically been based mainly on empirical breeder and trainer decisions rather than formal genetic evaluation. In practice, reining prospects are commonly chosen based on pedigree, earnings, conformation, athletic ability, responsiveness, trainability, and behavioral attributes related to willingness and consistency under training and competition.

Each horse–rider pair executes a prescribed sequence of maneuvers that must be followed exactly as written, with judging beginning upon entry into the arena and ending upon completion of the final maneuver. These patterns consist of combinations of fundamental movements, including circles of varying size and speed, flying lead changes, spins, sliding stops, rollbacks, backups, and hesitations, arranged in predefined sequences. Performance is evaluated using a standardized scoring system in which a score of 70 represents an average run, and each maneuver is scored on a scale from −1.5 to +1.5 in 0.5‐point increments according to correctness and degree of difficulty. The final score is derived by adjusting the base score of 70 with maneuver scores and applicable penalties for deviations from the prescribed pattern or execution faults. Judges evaluate performances based on criteria such as smoothness, precision, and control, with particular emphasis on the horse being willingly guided with little or no apparent resistance. Scores are assigned by officially approved judges, and results are determined after each individual performance. Reining performances consist of a single continuous execution of the prescribed pattern. Thus, in the original reining scoring scale, higher final scores indicate better performance.

### 2.2 Phenotype Definition

According to ANCR/NRHA regulations, distinct competition formats lead to systematic differences in score aggregation. In official events (e.g., Futurity, Derby, Super Stakes, and Copa Sacramento), performances are evaluated by five judges, with the highest and lowest scores discarded and the final score corresponding to the sum of the remaining three judges' scores. In contrast, affiliated events may involve a variable number of judges. Consequently, observed raw scores exhibit structural heterogeneity in scale across competitions, reflecting differences in the number of judges and aggregation procedures rather than solely biological variation in performance. Precise information on the number of judges per event was not available in the dataset. To address this limitation, alternative phenotypic definitions were derived within each contemporary group (defined as event–date–class combinations). First, a within‐group ranking (RANK) was computed based on the relative position of each performance. Second, a normalized score (BLOM) was obtained by applying a Blom transformation to the within‐group ranks. For both RANK and BLOM, lower values indicate better within‐group performance. BLOM was defined as follows:

$$y_{\mathrm{ij}}=\Phi^{-1}\left(\frac{r_{\mathrm{ij}}-0.375}{n_{j}+0.25}\right),$$

where $y_{\mathrm{ij}}$ is the Blom‐transformed score of the ith individual in group j; $r_{\mathrm{ij}}$ is the rank of the ith individual within group j; $n_{j}$ is the number of observations in group j; and $\Phi^{-1}$ denotes the inverse cumulative distribution function of the standard normal distribution. These transformations are invariant to linear scaling and preserve the relative ordering of performances within each group, thereby mitigating biases introduced by heterogeneous scoring scales. All phenotypic definitions (RANK and BLOM) were subsequently used in genetic analyses to evaluate their impact on variance components, genetic relationships across ages, and predictive ability.

### 2.3 Quality Control of Data

Based on an initial exploratory assessment, only records from horses aged between 30 and 144 months at the time of the event were retained. Records from youth competitor classes were excluded to ensure consistency in competitive conditions. Additionally, riders with fewer than three performances were removed, as were contemporary groups with fewer than five horses and horses with fewer than three observations, in order to guarantee sufficient data structure for reliable estimation of genetic parameters. Within ANCR/NRHA regulations, a score of 0 reflects a completed performance that is invalidated by major execution errors or loss of control and was therefore retained as a valid observation in our analyses, representing approximately 10% of the records. In contrast, a no score corresponds to disqualification due to rule violations unrelated to performance quality and was excluded from the dataset, accounting for approximately 5% of the original records. After applying these filtering criteria, 31% of the phenotypic records, 34% of the horses, and 8% of the events originally available were excluded, resulting in a final dataset comprising 210 events, 21,165 phenotypic records, and 1603 horses. Descriptive statistics of Quarter Horse reining competition data after quality control are presented in Table 1.

**TABLE 1:** Descriptive statistics of Quarter Horse reining competition data considering different phenotypic definitions: final score (SCORE), within‐group rank (RANK), and Blom‐transformed rank (BLOM).

Statistics	Value
Horses in the pedigree file, *n*	12,692
Records, *n*	21,165
Horses with records, *n*	1603
Mean number of records per horse, *n*	13.18
Horses with up to 10 records, *n*	991
Horses with 11 to 20 records, *n*	302
Horses with 21 to 30 records, *n*	150
Horses with 31 to 40 records, *n*	76
Horses with 41 to 50 records, n	36
Horses with more than 50 records, *n*	51
Male records, *n*	15,099
Female records, *n*	6066
Stallions with progeny record, *n*	290
Mares with progeny record, *n*	998
Contemporary groups (event–date–class), *n*	1532
Riders, *n*	582
Mean (SD) SCORE	137.35 (78.77)
Minimum and maximum SCORE	0 to 231
Records with SCORE = 0, *n*	2414
Mean (SD) RANK	10.73 (10.75)
Minimum and maximum RANK	1 to 68
Mean (SD) BLOM	0.00 (0.90)
Minimum and maximum BLOM	−2.28 to 2.37

Abbreviation: SD = standard deviation.

### 2.4 Models

(Co)variance components and genetic parameters were estimated separately for RANK and BLOM using a repeatability animal model and RRM of increasing polynomial order (linear, quadratic, and cubic), fitted as a function of age of horses at competition. Annual age classes were used to describe the data structure and to present age‐specific estimates of genetic parameters. These classes were defined as follows: 3 years (30–47 months), 4 years (48–59 months), 5 years (60–71 months), 6 years (72–83 months), 7 years (84–95 months), 8 years (96–107 months), 9 years (108–119 months), and 10 years (120–144 months). The number of horses per age class was 1103, 969, 640, 449, 316, 230, 138, and 114 for ages 3 to 10 years, respectively. The corresponding numbers of phenotypic records were 4454, 5187, 3537, 2743, 2060, 1429, 850, and 905, respectively.

For each RRM, models were fitted including a regression of the same polynomial order for all effects (additive genetic and permanent environmental). In total, four models were evaluated, comprising one repeatability model and three random regression models. The evaluated models included the same basic structure and differed only in the order of the random regressions fitted for the additive genetic and animal permanent environmental effects. The rider effect was modeled as an uncorrelated random effect. The RRM can be described as follows:

$$y_{\mathrm{ikl}}=\sum_{m=0}^{3}\beta_{m}\phi_{m}\left(t\right)+\sum_{m=0}^{p}\alpha_{\mathrm{im}}\phi_{m}\left(t\right)+\sum_{m=0}^{p}\gamma_{\mathrm{im}}\phi_{m}\left(t\right)+r_{k}+f_{l}+e_{\mathrm{ikl}}$$

where $\left(y_{\mathrm{ikl}}\right)$ is the phenotype recorded for the *i*th horse, ridden by the *k*th rider, in the *l*th contemporary group‐sex class; $\left(\beta_{m}\right)$ is the *m*th fixed regression coefficient describing the population mean trajectory as a function of age at competition (*t*); $\left(\phi_{m}\left(t\right)\right)$ is the *m*th Legendre orthogonal polynomial evaluated at age (*t*), with *p* = 1, 2, or 3 corresponding to linear, quadratic, and cubic specifications, respectively; $\left(\alpha_{\mathrm{im}}\right)$ and $\left(\gamma_{\mathrm{im}}\right)$ are the random regression coefficients for the additive genetic and animal permanent environmental effects, respectively; $\left(r_{k}\right)$ is the uncorrelated random effect of the *k*th rider; $\left(f_{l}\right)$ represents the set of systematic effects, including contemporary group and sex; and $\left(e_{\mathrm{ikl}}\right)$ is the residual term. Residual variance was assumed to be homogeneous across ages based on a preliminary analysis of the data, which indicated only minor variation in this component.

The full (co)variance structure of the model was defined using the vectors of random regression coefficients for the additive genetic and animal permanent environmental effects. For animal (*i*), these vectors were defined as follows:

$$\alpha_{i}=\left[\begin{array}{c} \alpha_{i0}\\ \alpha_{i1}\\ \vdots \\ \alpha_{\mathrm{ip}} \end{array}\right]\text{and}\;\gamma_{i}=\left[\begin{array}{c} \gamma_{i0}\\ \gamma_{i1}\\ \vdots \\ \gamma_{\mathrm{ip}} \end{array}\right].$$

The covariance matrix of the additive genetic random regression coefficients was

$$G=\left[\begin{array}{cccc} \sigma_{\alpha_{0}}^{2} & \sigma_{\alpha_{0}\alpha_{1}} & \cdots & \sigma_{\alpha_{0}\alpha_{p}}\\ \sigma_{\alpha_{1}\alpha_{0}} & \sigma_{\alpha_{1}}^{2} & \cdots & \sigma_{\alpha_{1}\alpha_{p}}\\ \vdots & \vdots & \ddots & \vdots \\ \sigma_{\alpha_{p}\alpha_{0}} & \sigma_{\alpha_{p}\alpha_{1}} & \cdots & \sigma_{\alpha_{p}}^{2} \end{array}\right],$$

and the covariance matrix of the animal permanent environmental random regression coefficients was

$$P=\left[\begin{array}{cccc} \sigma_{\gamma_{0}}^{2} & \sigma_{\gamma_{0}\gamma_{1}} & \cdots & \sigma_{\gamma_{0}\gamma_{p}}\\ \sigma_{\gamma_{1}\gamma_{0}} & \sigma_{\gamma_{1}}^{2} & \cdots & \sigma_{\gamma_{1}\gamma_{p}}\\ \vdots & \vdots & \ddots & \vdots \\ \sigma_{\gamma_{p}\gamma_{0}} & \sigma_{\gamma_{p}\gamma_{1}} & \cdots & \sigma_{\gamma_{p}}^{2} \end{array}\right].$$

Thus, the distributions of the random effects were assumed to be

$$\begin{array}{c} \begin{array}{c} \alpha ∼N\left(0,A⊗G\right),\\ \gamma ∼N\left(0,I_{a}⊗P\right),\\ r∼N\left(0,I_{r}\sigma_{r}^{2}\right), \end{array}\\ e∼N\left(0,I_{n}\sigma_{e}^{2}\right), \end{array}$$

where $\left(\alpha \right)$ and $\left(\gamma \right)$ are the vectors of additive genetic and animal permanent environmental random regression coefficients for all animals; $\left(A\right)$ is the numerator relationship matrix among animals; $\left(I_{a}\right)$, $\left(I_{r}\right)$, and $\left(I_{n}\right)$ are identity matrices with dimensions equal to the number of animals, riders, and phenotypic records, respectively; $\left(\sigma_{r}^{2}\right)$ is the rider variance; $\left(\sigma_{e}^{2}\right)$ is the residual variance; and $\left(⊗\right)$ denotes the Kronecker product. All random effects were assumed to be mutually independent.

In matrix notation, the phenotypic covariance structure can be written as follows:

$$V=Z_{a}\left(A⊗G\right)Z_{a}^{\prime }+Z_{\mathrm{pe}}\left(I_{a}⊗P\right)Z_{\mathrm{pe}}^{\prime }+Z_{r}I_{r}\sigma_{r}^{2}Z_{r}^{\prime }+I_{n}\sigma_{e}^{2}$$

where $\left(Za\right)$ *,* $\left(Z\mathrm{pe}\right)$, and $\left(Z_{r}\right)$ are the incidence matrices relating phenotypic records to additive genetic random regression coefficients, animal permanent environmental random regression coefficients, and rider effects, respectively.

For a given age (*t*), let

$$\phi \left(t\right)=\left[\begin{array}{c} \phi_{0}\left(t\right)\\ \phi_{1}\left(t\right)\\ \vdots \\ \phi_{p}\left(t\right) \end{array}\right]$$

The additive genetic and animal permanent environmental variances at age (*t*) were obtained as follows:

$$\sigma_{a}^{2}\left(t\right)=\phi {\left(t\right)}^{\prime }G\phi \left(t\right)$$

and

$$\sigma_{\mathrm{pe}}^{2}\left(t\right)=\phi {\left(t\right)}^{\prime }P\phi \left(t\right).$$

The phenotypic variance at age (*t*) was therefore

$$\sigma_{y}^{2}\left(t\right)=\sigma_{a}^{2}\left(t\right)+\sigma_{\mathrm{pe}}^{2}\left(t\right)+\sigma_{r}^{2}+\sigma_{e}^{2}$$

Additive genetic and animal permanent environmental covariances between two ages, (*t*_*1*_) and (*t*_*2*_), were computed as follows:

$$\sigma_{a}\left(t_{1},t_{2}\right)=\phi {\left(t_{1}\right)}^{\prime }G\phi \left(t_{2}\right)$$

and

$$\sigma_{\mathrm{pe}}\left(t_{1},t_{2}\right)=\phi {\left(t_{1}\right)}^{\prime }P\phi \left(t_{2}\right).$$

Accordingly, correlations between ages were obtained by dividing each covariance by the square root of the product of the corresponding variances at the two ages.

(Co)variance components and genetic parameters were estimated under Bayesian approach using the GIBBSF90 + program (Misztal et al. 2014). Chains of 650,000 samples were generated, with a burn‐in period of 50,000 samples and a thinning interval of 50. Convergence was assessed by visual inspection of trace plots to evaluate chain mixing. The remaining 12,000 samples were used to compute posterior means and highest posterior density intervals at 95% (HPD95%) for the genetic parameters.

### 2.5 Model Comparison

The deviance information criterion (DIC) (Spiegelhalter et al. 2002) was used to evaluate and compare model fit within each phenotype, RANK and BLOM. The DIC combines a measure of overall goodness of fit, given by the posterior mean of the deviance, with a penalty term that accounts for model complexity. Lower DIC values indicate a better‐fitting model.

### 2.6 Breeding Values

Age‐specific estimated breeding values (EBVs) were derived for each horse from the additive genetic random regression coefficients estimated in the selected model. For horse *i* at age *t*, the EBV was computed as follows:

$${\hat{\mathrm{EBV}}}_{i}\left(t\right)=\phi {\left(t\right)}^{\prime }{\hat{\alpha }}_{i}$$

where $\left(\phi \left(t\right)\right)$ is the vector of Legendre orthogonal polynomials evaluated at age (*t*) and $\left({\hat{\alpha }}_{i}\right)$ is the vector of estimated additive genetic random regression coefficients for horse *i*. EBV were obtained for each evaluated age class from 3 to 10 years for both RANK and BLOM. Stallions with at least five progeny records were selected for this evaluation (*N* = 66). Their age‐specific EBV were used to describe changes in predicted genetic merit across ages at competition. Spearman rank correlations were also computed among age‐specific EBV of these stallions for RANK and BLOM to evaluate the consistency of sire ranking across ages. For these stallions, the theoretical accuracy of overall genetic merit was calculated from a total EBV obtained by summing age‐specific EBV across all evaluated ages:

$${\hat{\mathrm{EBV}}}_{i,\text{total}}=\sum_{t=3}^{10}{\hat{\mathrm{EBV}}}_{i}\left(t\right)={\left[\sum_{t=3}^{10}\phi \left(t\right)\right]}^{\prime }{\hat{\alpha }}_{i}=s^{\prime }{\hat{\alpha }}_{i}.$$

The additive genetic variance associated with total EBV was obtained from the covariance matrix of the additive genetic random regression coefficients as: $\sigma_{a,\text{total}}^{2}=s^{\prime }\mathrm{Gs}$. The prediction error variance of total EBV was approximated by the squared posterior standard deviation (SD) of $\left({\mathrm{EBV}}_{i,\text{total}}\right)$. The theoretical accuracy for stallion i was then calculated as follows:

$${\text{Accuracy}}_{i}=\sqrt{1-\frac{SD^{2}\left({\mathrm{EBV}}_{i,\text{total}}\right)}{\left(1+F_{i}\right)\sigma_{a,\text{total}}^{2}}},$$

where $F_{i}$ is the inbreeding coefficient of stallion *i*.

### 2.7 Genetic Trends

Genetic trends were evaluated using the posterior mean of total EBV obtained for each horse across ages and the age‐specific EBV at 3 and 8 years of age. These ages were selected to represent early‐ and later‐life performance stages, respectively. For each phenotype and EBV definition, EBV were standardized to a mean of zero and a standard deviation of one. This standardization was used to allow direct comparison of the magnitude of genetic trends between RANK and BLOM, which are measured on different scales. For each birth year, the annual mean standardized total EBV was calculated considering all animals born in that year. Only birth years with at least 50 animals were included (2002 to 2021). A simple linear regression model was then fitted using the annual mean standardized total EBV as the dependent variable and birth year as the independent variable. The significance of the regression slope was assessed to test the null hypothesis that the genetic trend was equal to zero.

### 2.8 Predictive Validation Using LR Statistics Under Alternative Data Partitioning Schemes

Predictive ability was assessed using LR statistics (Legarra and Reverter 2018) under two complementary data‐partitioning schemes designed to represent distinct validation contexts. In Scheme A, horses were classified based on the year of first competition, with those entering the system in 2023 or later defined as focal individuals whose phenotypes were masked in the partial dataset. This scheme represents a forward validation scenario, in which predictions are evaluated for recently introduced animals lacking own performance records. In Scheme B, horses were partitioned according to age, with records from animals aged ≥ 7 years defined as focal observations and therefore masked in the partial dataset, representing a later‐life performance context. To ensure comparability across phenotypes, only LR‐derived statistics based on the covariance structure between predictions from the whole and partial datasets were considered, namely dispersion (regression slope of whole on partial evaluations), ratio of accuracies (correlation between whole and partial predictions), and empirical reliability, defined as the covariance between whole and partial predictions divided by the variance of whole predictions. In this context, values close to 1 are expected for dispersion, indicating well‐calibrated predictions; for the ratio of accuracies, values approaching 1 indicate high agreement between evaluations; and for empirical reliability, higher values reflect greater predictive ability. These metrics were computed and reported across the full evaluated age range from 3 to 10 years for both validation schemes. However, interpretation of predictive performance was emphasized within the age intervals most consistent with the structure and objective of each validation scheme: ages 3 to 6 years for Scheme A and ages 7 to 10 years for Scheme B. This emphasis was necessary because, under Scheme A, the number of animals and records decreased substantially beyond 6 years of age, with focal animals representing approximately 33% of individuals and 26% of records, and about 95% of the validation phenotypes concentrated between 3 and 6 years of age. In contrast, under Scheme B, validation information was inherently concentrated at older ages due to the ≥ 7‐year cutoff, with focal records accounting for approximately 25% of the data and 27.5% of the animals, distributed across ages 7 to 10 years.

## 3 Results

### 3.1 Phenotypic Distributions

**FIGURE 1:**
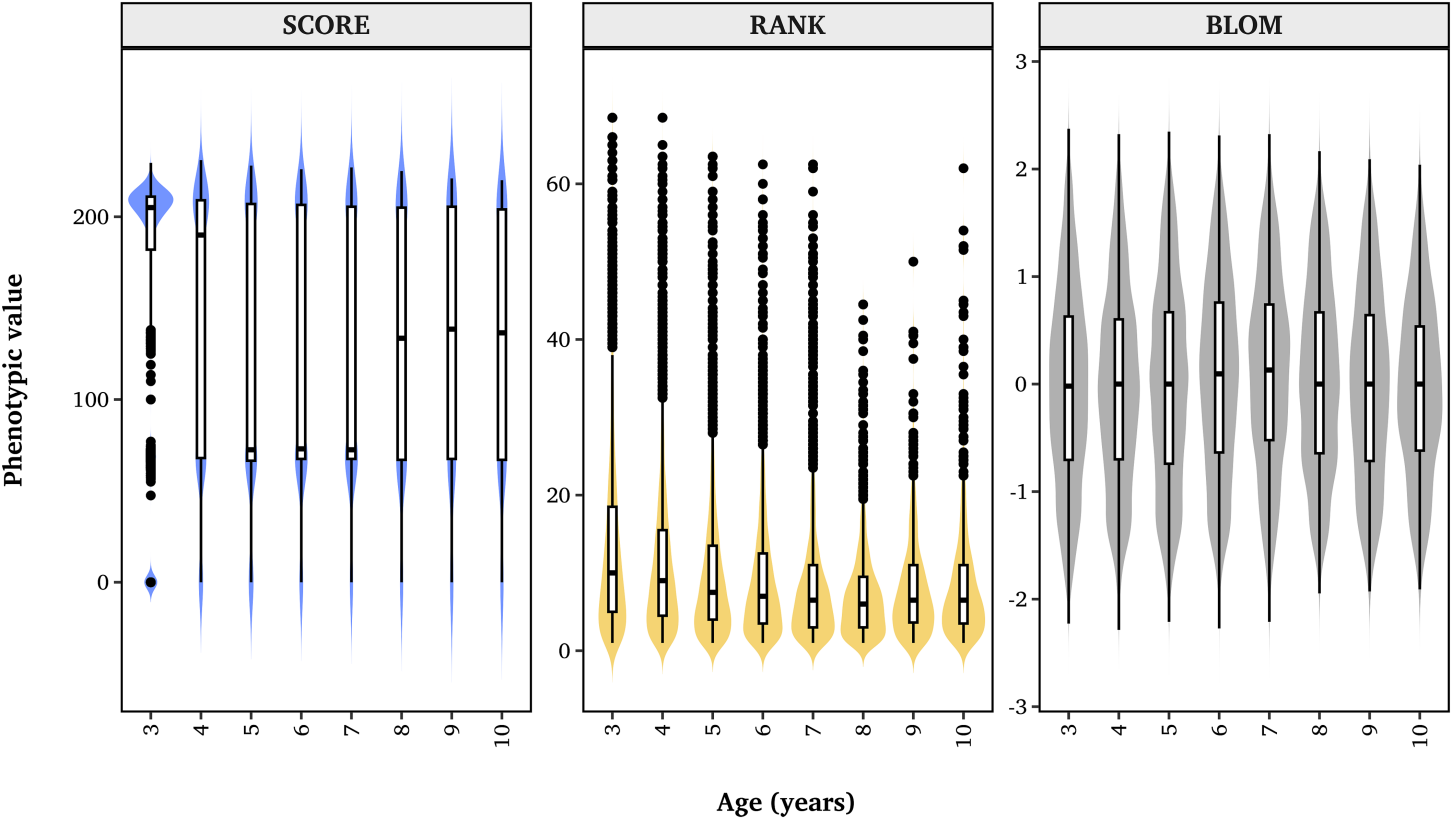
Distribution of phenotypes across age classes in reining Quarter Horses, presented as violin plots for final score (SCORE), within‐group rank (RANK), and Blom‐transformed rank (BLOM). Each violin represents the density of observations within age class, with vertical bars indicating the range of the data and horizontal lines denoting the median and interquartile range within each distribution.

The final score exhibited a heterogeneous distribution (Figure 1), reflecting variation in the number of judges and score aggregation procedures across competitions, as previously described. The distribution of phenotypic records differed markedly between RANK and BLOM, reflecting the underlying transformation applied within contemporary groups. As expected, RANK exhibited a discrete and right‐skewed distribution, with a higher frequency of lower rank values and variation in group sizes. In contrast, BLOM displayed a continuous and approximately symmetric distribution centered around zero, consistent with its normalization properties. The BLOM transformation effectively reduced heterogeneity in dispersion across groups, whereas RANK retained more pronounced variability associated with differences in group size, highlighting distinct statistical properties that may influence subsequent genetic analyses.

### 3.2 Model Comparison

Model comparison based on the DIC revealed a consistent improvement in model fit when moving from the repeatability model (order 0) to RRM for both phenotypes, RANK and BLOM (Table 2). The repeatability model showed the poorest fit, indicating that assuming constant genetic and environmental effects across ages was not adequate to capture the underlying longitudinal structure of performance. Increasing the order of the polynomial in the RRM also led to progressive improvements in model fit. The best fit was consistently achieved with cubic RRM, suggesting that third‐order polynomials were sufficient to adequately capture the trajectory of performance across ages. A higher‐order polynomial model (quartic RRM) was also evaluated; however, this specification resulted in severe fitting problems, likely due to overparameterization relative to the structure and size of the present dataset. Therefore, based on model fit and numerical stability, the cubic RRM was selected as the most appropriate model and was subsequently used to present genetic parameter estimates and breeding value trajectories for both phenotypes.

**TABLE 2:** Deviance information criterion (DIC) for alternative model specifications in the analysis of reining performance in Quarter Horses, considering different phenotypic definitions: within‐group rank (RANK) and Blom‐transformed rank (BLOM).

Phenotype	Model order	DIC	ΔDIC
RANK	0	225,246.2	1846.2
	1	224,590.7	1190.7
	2	224,075.0	675
	3	223,400.0	0
BLOM	0	236,940.5	1331.9
	1	236,427.7	819.1
	2	235,930.2	321.6
	3	235,608.6	0

*Note:* RR = random regression for rider effect. Model order = order of Legendre polynomial. ΔDIC = difference relative to the best model, defined as the lowest DIC within each phenotype.

### 3.3 Heritability, Repeatability and Rider Effect

**FIGURE 2:**
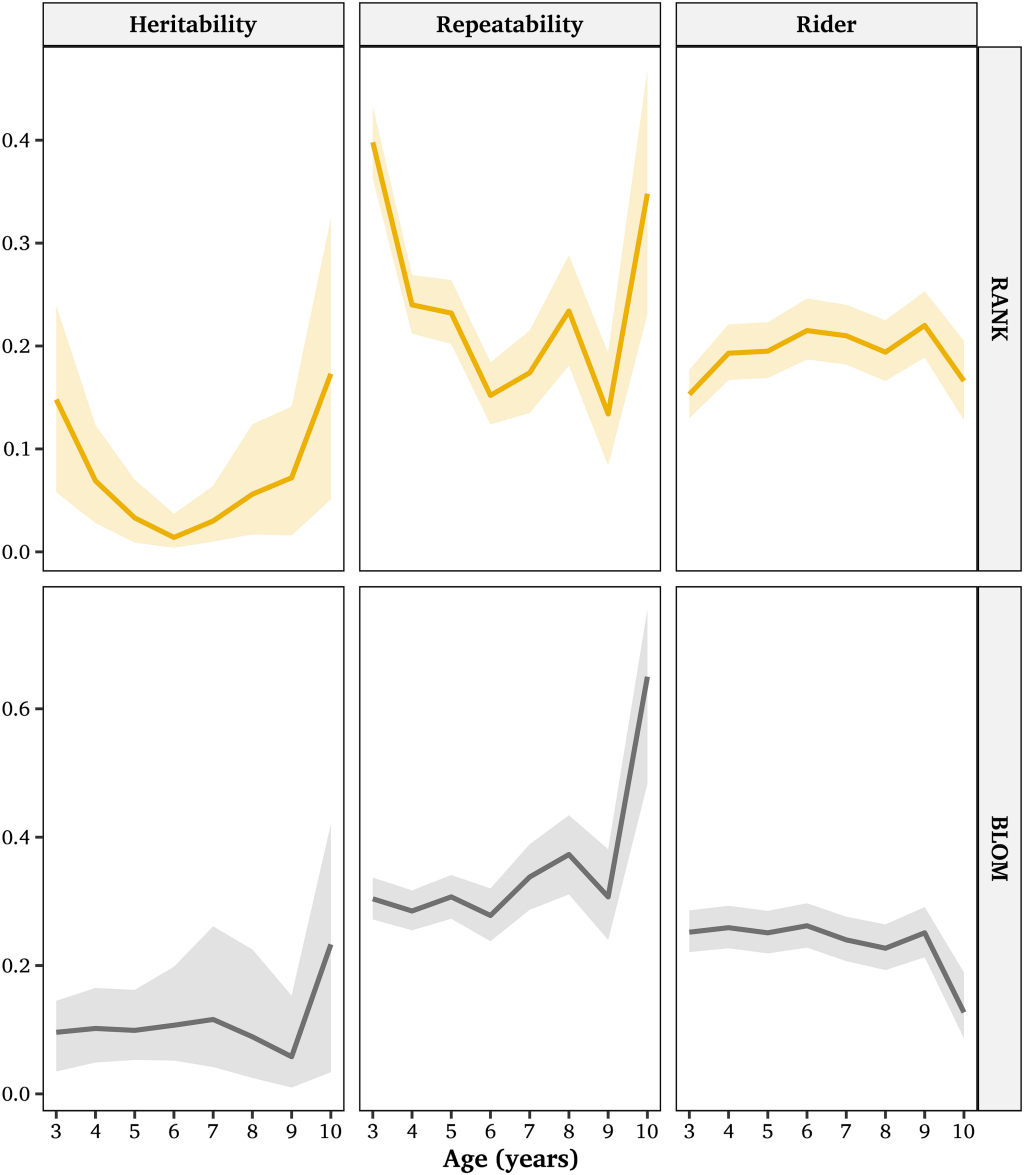
Posterior means (lines) and 95% highest posterior density intervals (shaded areas) for heritability, repeatability, and rider variance (expressed as a proportion of phenotypic variance) across ages in reining Quarter Horses, presented separately for the RANK and BLOM phenotypes.

Posterior mean heritability estimates were generally low and varied across ages for both phenotypes, RANK and BLOM (Figure 2), indicating age‐dependent changes in the contribution of additive genetic variance to performance. The posterior estimates of variance components are provided in Supporting Information S2 and further show heterogeneous additive genetic variances across ages for reining performance. For RANK, heritability was higher at 3 years (0.15; HPD95%: 0.06–0.24), declined to lower values between 4 and 6 years (e.g., 0.01 at 6 years; HPD95%: 0.00–0.04), and increased again at older ages, reaching 0.17 at 10 years (HPD95%: 0.05–0.32). For BLOM, heritability remained low to moderate from 3 to 9 years, with posterior means ranging from 0.06 to 0.12. Estimates were relatively stable from 3 to 7 years, followed by lower values at 8 and 9 years. The highest estimate was observed at 10 years (0.23; HPD95%: 0.07–0.40), although with a wider credible interval.

Permanent and rider effects accounted for a considerable proportion of the phenotypic variance across ages for both phenotypes, with distinct patterns observed for RANK and BLOM. For BLOM, repeatability remained moderate and relatively stable from 3 to 9 years (posterior means around 0.31; HPD95%: 0.27–0.36), followed by a marked increase at 10 years (0.65; HPD95%: 0.48–0.75). The proportion of variance explained by rider effects was also moderate and consistent across most ages (e.g., 0.26 at 7 years; HPD95%: 0.23–0.30), with a reduction at 10 years (0.13; HPD95%: 0.09–0.19). In contrast, RANK showed higher repeatability at younger ages, peaking at 3 years (0.40; HPD95%: 0.36–0.43), followed by a decline to lower values between 6 and 9 years (e.g., 0.17 at 7 years; HPD95%: 0.14–0.22). Rider‐related variance for RANK was consistently lower, remaining around 0.19 (HPD95%: 0.16–0.22).

### 3.4 Genetic Correlation

**FIGURE 3:**
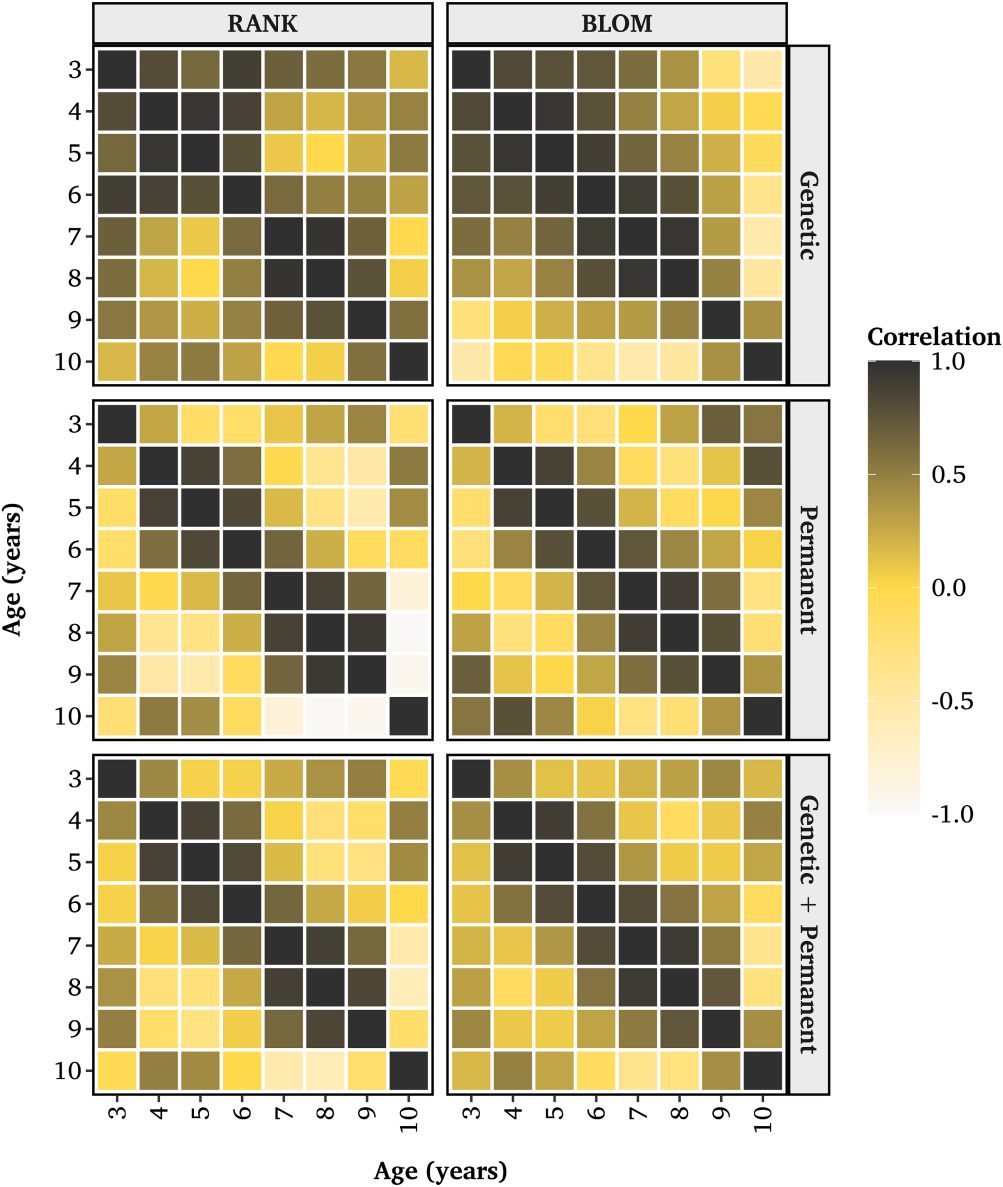
Posterior means of additive genetic, permanent environmental, and combined additive genetic + permanent environmental correlations across ages in reining Quarter Horses, estimated for within‐group rank (RANK) and Blom‐transformed rank (BLOM).

Correlations across ages differed between RANK and BLOM (Figure 3). Overall, mean correlations across all age combinations were 0.50 for additive genetic effects, 0.10 for permanent environmental effects, and 0.22 for the combined additive genetic + permanent environmental effect for RANK. For BLOM, the corresponding mean correlations were 0.36, 0.30, and 0.32, respectively. For RANK, additive genetic correlations were generally positive, with high estimates between several adjacent ages, including 4 and 5 years (0.96; HPD95%: 0.87–0.99) and 7 and 8 years (0.97; HPD95%: 0.89–1.00). Lower estimates were observed for some distant age comparisons, particularly those involving 10 years, such as between 7 and 10 years (−0.03; HPD95%: −0.51–0.47). BLOM also showed high additive genetic correlations between adjacent ages, including 4 and 5 years (0.96; HPD95%: 0.89–1.00) and 6 and 7 years (0.92; HPD95%: 0.79–0.98). However, correlations decreased for more distant age pairs and became negative in some comparisons involving 10 years, such as between 7 and 10 years (−0.54; HPD95%: −0.71 to −0.26). Permanent environmental correlations were more variable than additive genetic correlations for both phenotypes. For RANK, high positive correlations were observed between some adjacent ages, such as 7 and 8 years (0.88; HPD95%: 0.81–0.92), whereas strong negative estimates were observed for comparisons involving 10 years, especially between 9 and 10 years (−0.94; HPD95%: −0.98 to −0.86). For BLOM, permanent environmental correlations were generally positive among adjacent ages, including 4 and 5 years (0.88; HPD95%: 0.83–0.92) and 7 and 8 years (0.91; HPD95%: 0.80–0.95), but lower or negative estimates were observed for some distant age comparisons.

Correlations for the combined additive genetic + permanent environmental effect showed intermediate patterns (Figure 3). For RANK, high positive correlations were observed between 7 and 8 years (0.89; HPD95%: 0.85–0.93), whereas negative estimates occurred for some comparisons involving later ages, such as 7 and 10 years (−0.55; HPD95%: −0.74 to −0.21). For BLOM, the combined‐effect correlations were also high between adjacent ages, including 5 and 6 years (0.82; HPD95%: 0.77–0.87), and 7 and 8 years (0.93; HPD95%: 0.89–0.95), but decreased for more distant age pairs and were negative for 7 and 10 years (−0.36; HPD95%: −0.57 to −0.16).

### 3.5 Estimated Breeding Values and Rank Across Ages

**FIGURE 4:**
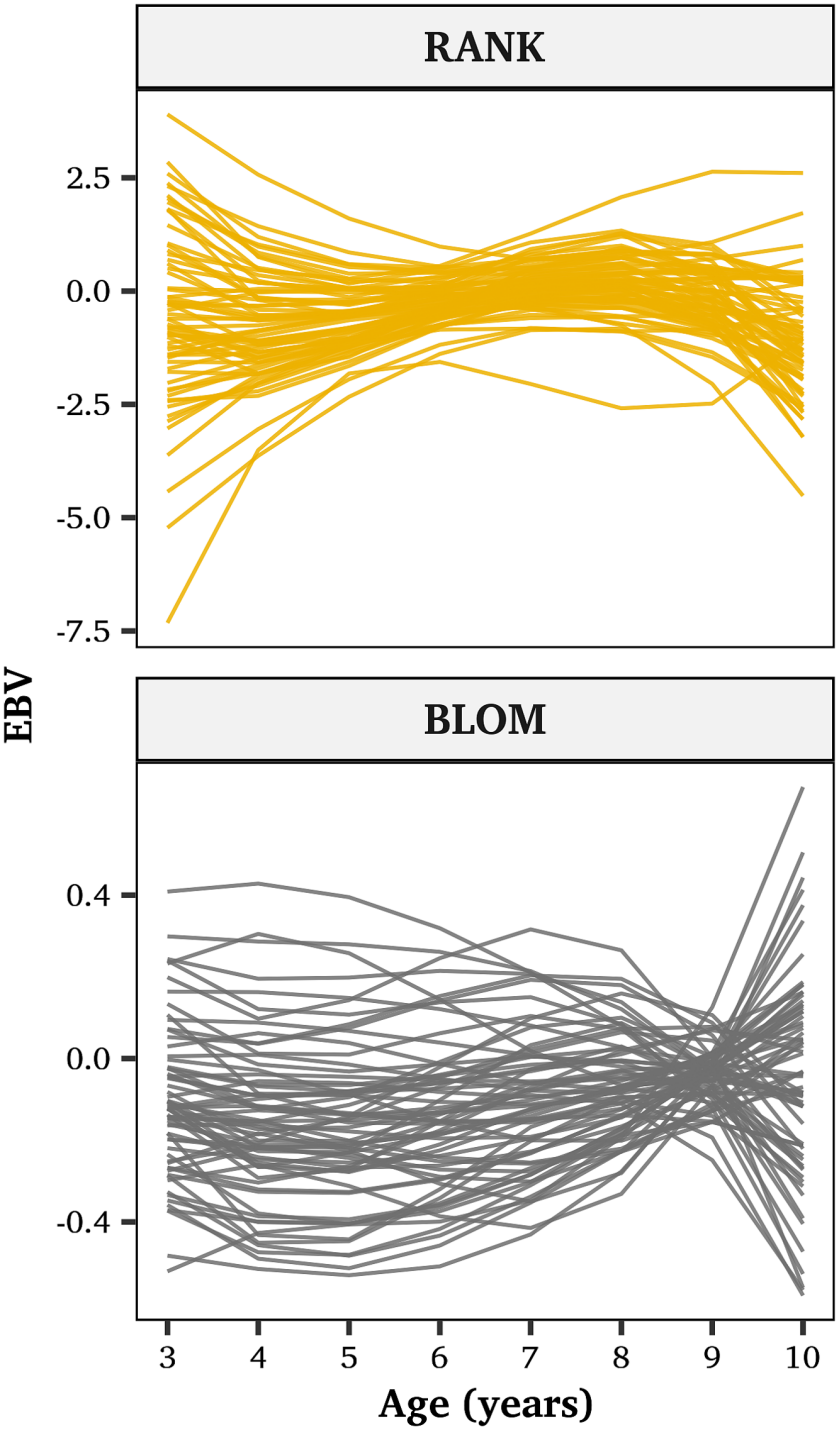
Posterior means of estimated breeding values (EBV) for reining performance in Quarter Horse stallions across ages at competition, estimated for within‐group rank (RANK) and Blom‐transformed rank (BLOM).

Figure 4 presents EBV trajectories for RANK and BLOM based on a sample of 66 stallions with at least five progeny records. Substantial changes in EBV were observed across ages. Spearman correlations between RANK and BLOM EBV for this group of stallions were strong and significant (*p* < 0.01) at younger ages, with values of 0.89, 0.91, 0.90, 0.85, 0.71, and 0.62 at 3, 4, 5, 6, 7, and 8 years, respectively. However, correlations were not significantly different from zero (*p* > 0.01) at older ages, with estimates of 0.06 and 0.26 at 9 and 10 years, respectively. The Spearman correlation between total EBV for RANK and BLOM, computed as the sum of EBV across all ages for each animal, was 0.82 (*p* < 0.01).

Theoretical accuracy of total EBV was moderate for both phenotypes among the 66 selected stallions. RANK showed slightly higher accuracy than BLOM, with a mean of 0.65 (SD = 0.10), median of 0.66, and values ranging from 0.43 to 0.85. For BLOM, the mean accuracy was 0.61 (SD = 0.12), with a median of 0.62 and a range from 0.26 to 0.81. The interquartile range was 0.57 to 0.71 for RANK and 0.54 to 0.69 for BLOM.

### 3.6 Genetic Trends

**FIGURE 5:**
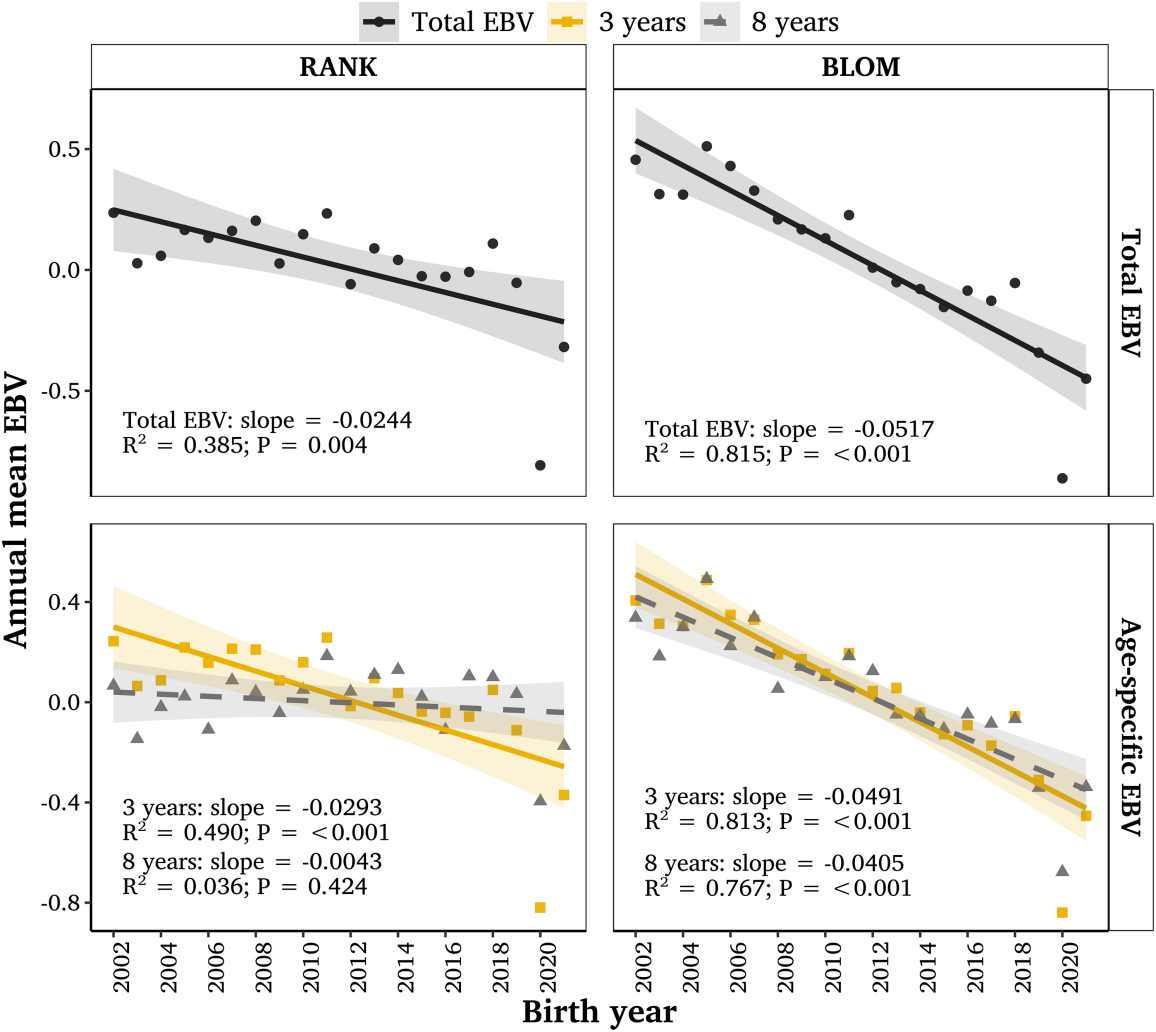
Genetic trends for total and age‐specific estimated breeding values (EBV) across birth years in reining Quarter Horses for within‐group rank (RANK) and Blom‐transformed rank (BLOM) phenotypes. Upper panels show trends for total EBV, whereas lower panels show trends for age‐specific EBV at 3 and 8 years of age. Annual means were standardized within each EBV definition and phenotype. *R* ^2^ denotes the coefficient of determination, slope denotes the regression coefficient from the linear model fitted to annual mean standardized EBV, and *p* denotes the significance level of the linear trend.

Genetic trends based on annual mean standardized total EBV showed a decreasing pattern across birth years for both phenotypes (Figure 5). For RANK, the standardized regression coefficient was −0.0244 EBV standard deviations per year (standard error = 0.0073; *p* = 0.004). For BLOM, the corresponding coefficient was −0.0517 EBV standard deviations per year (standard error = 0.0058; *p* < 0.001). Age‐specific genetic trends showed different patterns between phenotypes. For RANK, the trend at 3 years of age was negative and significant, with a standardized regression coefficient of −0.0293 EBV standard deviations per year (standard error = 0.0070; *p* < 0.001), whereas the trend at 8 years was close to zero and not significant (−0.0043 EBV standard deviations per year; standard error = 0.0052; *p* = 0.424). For BLOM, negative and significant trends were observed at both 3 and 8 years of age, with standardized regression coefficients of −0.0491 (standard error = 0.0055; *p* < 0.001) and −0.0405 EBV standard deviations per year (standard error = 0.0053; *p* < 0.001), respectively.

### 3.7 Predictive Validation Using LR Statistics

**FIGURE 6:**
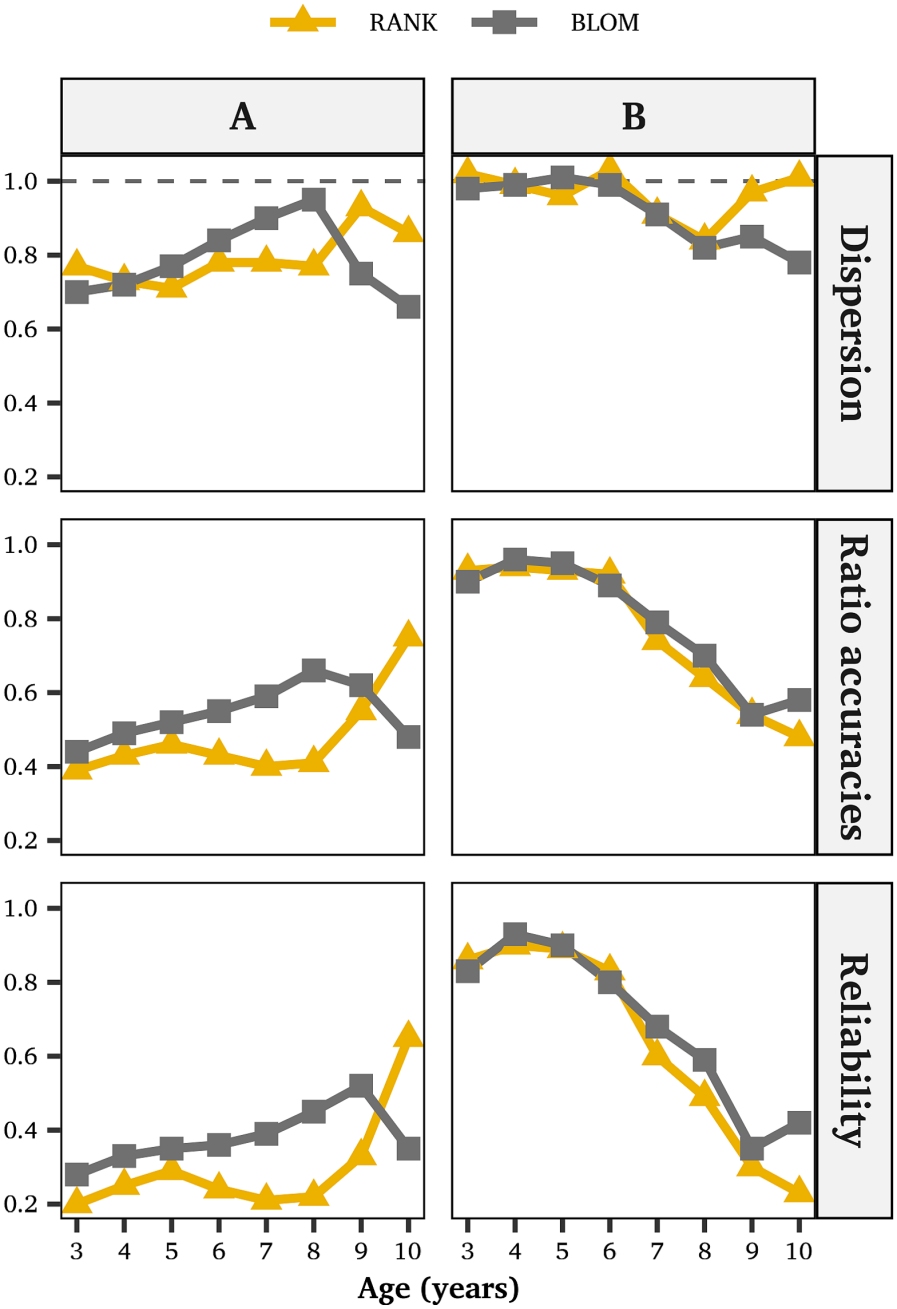
LR validation statistics across ages for within‐group rank (RANK) and Blom‐transformed rank (BLOM) under Scheme A and Scheme B. Dispersion measures calibration, with values close to 1 indicating unbiased predictions; ratio of accuracies measures agreement between whole‐ and partial‐data estimated breeding values; and empirical reliability indicates predictive ability, with higher values denoting more reliable predictions. In Scheme A, horses entering competition in 2023 or later were treated as focal individuals; in Scheme B, records from animals aged ≥ 7 years were treated as focal observations.

Under Scheme A, defined according to year of first competition, predictive validation showed age‐dependent patterns for both phenotypes (Figure 6). For RANK, dispersion remained relatively stable across most ages, with higher values at later ages, whereas the ratio of accuracies and empirical reliability increased more clearly toward 10 years, reaching 0.75 and 0.65, respectively. For BLOM, dispersion increased progressively up to 8 years and then declined at older ages. Compared with RANK, BLOM generally showed higher ratios of accuracies and empirical reliabilities from 3 to 9 years, with maximum values of 0.66 and 0.52, respectively. At 10 years, however, RANK showed the highest values for both statistics.

Under Scheme B, defined according to age‐based validation, both phenotypes showed favorable dispersion estimates at younger ages, with values close to 1.00. RANK showed slightly better dispersion at later ages, particularly from 8 to 10 years, whereas BLOM showed dispersion values closer to 1.00 mainly at intermediate ages. The ratio of accuracies and empirical reliability was generally high from 3 to 6 years for both phenotypes and declined at older ages. Overall, BLOM showed higher ratios of accuracies and empirical reliabilities across most ages, with peak values of 0.96 and 0.93, respectively, at 4 years. In contrast, RANK showed slightly higher values for these statistics at some specific ages, especially at 3 and 6 years, but declined more sharply by 10 years, reaching 0.48 for the ratio of accuracies and 0.23 for empirical reliability.

## 4 Discussion

Western performance disciplines, particularly reining, represent a rapidly expanding segment of the equine industry, combining athletic precision with substantial economic and breeding relevance. Despite their importance, genetic studies in these disciplines remain limited, leaving only a limited understanding of the genetic architecture underlying performance. To our knowledge, this study provides the first comprehensive estimation of genetic parameters for reining performance, offering novel insights into the inheritance patterns of this trait and establishing a foundation for future genetic evaluation and selection strategies in Quarter Horses.

The central objective of this study was to evaluate alternative phenotypic definitions for genetic evaluation of reining performance, a trait inherently characterized by non‐normality, subjectivity, and competition‐dependent structure. In equine genetic studies, transformations of original measures such as scores or rankings are commonly adopted to better align data with the assumptions of linear mixed models (Mezei et al. 2015; Thiruvenkadan et al. 2009). Among these, rank‐based approaches, particularly the Blom transformation, have been widely used due to their ability to approximate normality and improve statistical stability (Mezei et al. 2015; Novotná et al. 2014; Posta et al. 2009). However, these transformations inherently remove information on the magnitude of differences among individuals, preserving only relative positions (Cervantes et al. 2020). This limitation may be of limited practical concern in scenarios where the original scale is already affected by heterogeneity or subjectivity, as is often the case in judged performance traits. In addition, rank‐based phenotypes exhibit desirable statistical properties, including reduced sensitivity to unequal group sizes and increased robustness to highly skewed distributions (Röhe et al. 2001). Preliminary analyses using the untransformed raw score were also performed. However, these models showed convergence problems and biologically inconsistent estimates, including unrealistically high heritability with wide HPD95% intervals, particularly at older ages. These results were likely related to structural heterogeneity in the raw scoring scale across competitions. Therefore, raw score was not retained in the final analyses, and within‐group rank‐based phenotypes were considered more appropriate to mitigate scale heterogeneity while preserving the relative ordering of performances.

Importantly, the literature emphasizes that phenotype definition should not be guided solely by distributional considerations, but also by the resulting genetic parameter estimates and predictive performance of the models (García‐Ballesteros et al. 2018). Different transformations may yield comparable heritability or repeatability estimates while differing substantially in model adequacy and predictive ability, indicating that the choice of phenotype ultimately depends on its suitability for accurate genetic inference and selection decisions. Although the Blom transformation is widely used in genetic studies, its application should not be regarded as universally optimal. Beasley et al. (2009) emphasized that rank‐based inverse normal transformations do not necessarily normalize model residuals, may alter the null hypothesis being tested, and can yield undesirable statistical properties, including inadequate Type I error control and loss of power under some conditions, particularly in more complex models involving heterogeneous variances or interaction terms. Their conclusions therefore suggest that the usefulness of Blom‐type transformations is context‐dependent and should be judged empirically from the behavior of the fitted models rather than assumed a priori.

For the Quarter Horse population evaluated in this study, reining performance exhibited low heritability, with slightly higher estimates when final scores were transformed using the Blom approach. The estimates obtained herein fall within the range commonly reported in the literature for other traits in sports horses, including 0.02–0.07 for show‐jumping performance (Mezei et al. 2015), 0.09–0.19 (Chapard et al. 2025), and 0.06 to 0.14 for race time, 0.09–0.15 for placing, and 0.07–0.17 for rank in endurance horses across different breeds (Cervantes et al. 2020). These results indicate that, although modest, genetic gain through selection for reining performance is feasible. However, given the dynamic pattern observed for heritability and other genetic parameters across ages, responses to selection are expected to vary substantially depending on the stage of evaluation. This age‐dependent behavior is consistent with findings reported by Posta et al. (2010), who applied random regression models to shifted Blom normalized ranks and the difference between fence height and fault points in show‐jumping performance, observing heritability estimates ranging from 0.03 to 0.38 across ages.

Repeatability estimates obtained in this study were slightly lower, whereas the proportion of phenotypic variance explained by rider effects was very similar to that reported by Santana et al. (2025) for barrel racing performance in Quarter Horses in Brazil. Barrel racing is a highly popular discipline in countries where the Quarter Horse breed is well established and requires not only speed but also precise control, coordination, and substantial rider–horse skill to complete the course without penalties. Although reining competitions are not based on speed or explosive performance, riders must accurately guide and control their horses throughout a predefined pattern, executing each maneuver with strict precision. Therefore, it was expected that rider effects would account for a substantial proportion of the phenotypic variance in reining performance. The substantial rider effect observed here is consistent with findings by Gómez et al. (2010) in Spanish Trotter horses. These authors also noted that accumulated experience with age may reduce the relative importance of rider effects while increasing the contribution of permanent environmental effects. Our results are consistent with those reported for show jumping performance, for which Chapard et al. (2023) demonstrated that including rider effects can improve model fit in Warmblood horses. However, both Tennah et al. (2012) and Chapard et al. (2023) emphasized that rider effects can be difficult to disentangle when the number of horses per rider is limited or when riders are strongly associated with specific horses, leading to potential confounding with animal merit. Although such confounding cannot be entirely ruled out in the present study, it was mitigated by restricting the dataset to horses and riders with at least three recorded performances.

Another aspect that should be considered is the potential occurrence of genotype‐by‐sex interaction for reining performance. Although sex was included as a systematic effect in the present models, sex‐specific genetic parameters were not estimated. Previous studies in horses indicate that sex‐related differences may affect performance or the expression of genetic effects. Stewart et al. (2010) reported important differences between stallions and geldings for dressage performance, with stallions performing better, whereas Tozaki et al. (2012) showed sex‐specific associations between SNP genotypes and racing performance indicators in Japanese Thoroughbreds, with stronger genotype associations in females than in males. These findings suggest that males, females, and geldings may differ in physiological maturity, management, training intensity, reproductive use, and competitive opportunity, which could influence the expression of genetic merit across ages. Therefore, the possibility that genetic trajectories differ by sex cannot be ruled out. However, fitting sex‐specific random regression models would require substantially larger datasets, particularly because the number of records decreases at older ages.

Correlation estimates provided an additional criterion to assess the consistency of RANK and BLOM across ages. As expected, correlations for additive genetic, permanent environmental, and rider effects were higher between adjacent ages and declined as the age interval increased, in agreement with previous studies on longitudinal traits in sport horses (Bugislaus et al. 2006; Gómez et al. 2010; Santana et al. 2025). For additive genetic effects, RANK showed a higher overall mean correlation than BLOM and generally positive estimates across the age trajectory, suggesting greater genetic stability for this phenotype. In contrast, BLOM showed lower additive genetic consistency across distant ages, with negative estimates in some comparisons involving 10 years, indicating greater re‐ranking of EBV at later ages. However, for both phenotypes, correlations involving older ages were associated with wide HPD95% intervals, indicating considerable uncertainty, likely related to limited data support at the end of the age trajectory, a well‐recognized issue in random regression models (Oliveira et al. 2019). It is also important to consider that the reduced number of records at older ages may reflect a selection process within the competitive population. Horses with declining performance over time, whether due to aging, injuries, training limitations, or reduced competitive potential, may be less likely to remain active in competition at advanced ages. Therefore, estimates involving older age classes should be interpreted with caution, as they may be affected not only by reduced data support but also by selective participation of horses that remained competitive for longer periods.

For permanent environmental effects and for the combined additive genetic + permanent environmental effect, BLOM showed slightly higher overall mean correlations than RANK, indicating greater consistency for the non‐residual repeatable component of performance. Therefore, the relative stability of the phenotypes depended on the source of variation considered. Consistent with this pattern, Spearman correlations between sire EBV derived from RANK and BLOM were moderate to high at younger ages (≤ 8 years) but not different from zero at older ages, reinforcing the divergence between phenotype definitions as age increases and the number of records declines.

The genetic trends based on annual mean standardized total EBV were negative for both phenotypes, indicating favorable cumulative genetic change toward lower EBV values, which correspond to better competitive rankings. The use of standardized EBV allowed direct comparison between RANK and BLOM, and the more pronounced standardized slope observed for BLOM suggests a stronger favorable temporal change for this phenotype. The higher coefficient of determination for BLOM also indicates a more consistent linear pattern across birth years. Age‐specific trends further showed that genetic change was not uniform across ages. For RANK, the favorable trend was evident at 3 years but not at 8 years, whereas BLOM showed favorable trends at both ages. This pattern is consistent with the age‐dependent genetic structure observed in the random regression analyses and supports the relevance of evaluating genetic merit at different stages of the competitive trajectory. Together, these findings suggest that, despite the absence of a formal genetic evaluation program, historical empirical selection based on pedigree, conformation, training response, and competition results may have contributed to gradual genetic improvement for reining performance. However, because both phenotypes are rank‐based and depend on the competitive structure of each contemporary group, these trends should be interpreted as evidence of directional change in relative genetic merit rather than as direct change in an absolute performance scale.

Predictive validation based on LR statistics revealed that the relative performance of RANK and BLOM depended on the validation scheme, age class, and statistic considered. This indicates that the usefulness of a phenotype for genetic evaluation depends not only on its distributional properties but also on how well it preserves the competitive structure of the data and supports stable prediction across ages. In this context, Ricard and Legarra (2010) emphasized that rank‐based criteria analyzed on the observed scale may underestimate genetic variance and heritability due to scale limitations and model misspecification, particularly in structured competitions, and that transformations such as normal scores do not fully overcome these limitations. Complementarily, García‐Ballesteros et al. (2018) showed that models explicitly accounting for the competitive structure among individuals within events, such as the Thurstonian approach, can achieve higher predictive ability for ranking traits than linear or threshold models. In the present study, both phenotypes provided useful predictive information, but with different strengths. Under Scheme A, BLOM generally showed higher ratios of accuracies and empirical reliabilities from 3 to 9 years, whereas RANK reached the highest values for these statistics at 10 years. Under Scheme B, both phenotypes showed favorable dispersion estimates at younger ages, with values close to 1.00. BLOM showed higher ratios of accuracies and empirical reliabilities across most ages, especially at early and intermediate ages, whereas RANK showed slightly better dispersion at later ages and higher values for some specific ages. These results suggest that BLOM may provide better predictive performance for several age classes, whereas RANK may offer slightly more stable calibration in some later‐age comparisons. The theoretical accuracy of total EBV also supported a balanced interpretation: RANK showed only slightly higher mean accuracy than BLOM among selected stallions, with overlapping distributions between phenotypes. From a modeling perspective, although the Thurstonian approach provides a theoretically appealing framework for ranking traits (Cervantes et al. 2020; Gama et al. 2016; Varona and Legarra 2020), it was not considered here, and its extension to longitudinal random regression models remains limited. In contrast, linear models remain a practical and flexible alternative for longitudinal genetic evaluation, even for non‐normally distributed or ranking‐derived traits, and their empirical performance has been shown to be competitive or superior in longitudinal settings. In particular, linear random regression models have outperformed threshold approaches even for binary traits (Jamrozik et al. 2013), while offering greater flexibility to model time‐dependent effects (Oliveira et al. 2019).

From a practical perspective, these findings support the development of a formal genetic evaluation strategy for reining performance in the Brazilian Quarter Horse population. Because heritability was low but additive genetic variation was present, future genetic progress will likely depend on the systematic accumulation of standardized competition records, improved pedigree completeness, and routine estimation of EBV. In this context, BLOM may be useful as a normalized ranking‐derived phenotype for routine evaluation, whereas RANK can serve as a complementary descriptor of relative competitive outcomes. Future breeding programs should also prioritize stronger connectedness among contemporary groups, consistent recording of rider and competition information, and systematic registration of the number of judges and scoring procedures used in each event. In the longer term, the integration of genomic information may further improve accuracy, particularly for young horses with limited own performance records.

This study showed that reining performance in Brazilian Quarter Horses can be modeled using random regression models, allowing age‐specific estimation of genetic parameters and breeding values. Heritability estimates were generally low but indicated the presence of additive genetic variation, supporting the feasibility of genetic selection for reining performance. The results also demonstrated that phenotypic definition influences genetic inference, EBV trajectories, genetic trends, and predictive validation. RANK and BLOM provided broadly comparable and complementary phenotype definitions, each with specific strengths. BLOM showed modest advantages for several criteria, including slightly higher heritability estimates, greater consistency for the combined additive genetic + permanent environmental component, stronger favorable genetic trends, and better LR‐based predictive performance across several age classes. In contrast, RANK showed slightly greater additive genetic stability across ages, marginally higher theoretical accuracy for total EBV among selected stallions, and favorable calibration in some later‐age comparisons. However, the differences between phenotypes were generally small and do not indicate clear superiority of one phenotype over the other. Thus, BLOM may be useful as a normalized ranking‐derived phenotype for routine evaluation, whereas RANK remains a relevant complementary measure for representing relative competitive performance on the observed scale. Overall, rank‐based phenotypes provide a practical strategy for genetic evaluation of reining performance, particularly in judged competition systems characterized by heterogeneous scoring procedures and contemporary group‐dependent outcomes.

## Funding

The authors have nothing to report.

## Conflicts of Interest

The authors declare no conflicts of interest.

## Supporting information


**Data S1:** Schematic representation of three official reining patterns illustrating typical maneuver sequences performed in competition. Diagrams indicate the relative positioning of arena markers (center and side markers at ~15 m from end walls), directional arrows representing horse movement, and standardized elements such as spins, circles of varying speed, sliding stops, rollbacks, and lead changes.

**Data S2:** Posterior summaries of variance components by age for within‐group rank (RANK) and Blom‐transformed rank (BLOM) of reining performance in Quarter Horses. Values are posterior means, standard deviations (SD), and 95% highest posterior density intervals (HPD95%) for rider, additive genetic, permanent environmental, and residual variance components obtained from a cubic random regression model.

## Data Availability

The data that support the findings of this study are available from the corresponding author upon reasonable request.
