# Supplementary figures and images for: Phenotypic Definition Influences Genetic Inference and Predictive Ability for Reining Performance in Quarter Horses Using Random Regression Models
Source: Anim Sci J. 2026 Jul 23;97(1):e70223. doi: 10.1111/asj.70223 (PMC13392984; doi:10.1111/asj.70223)

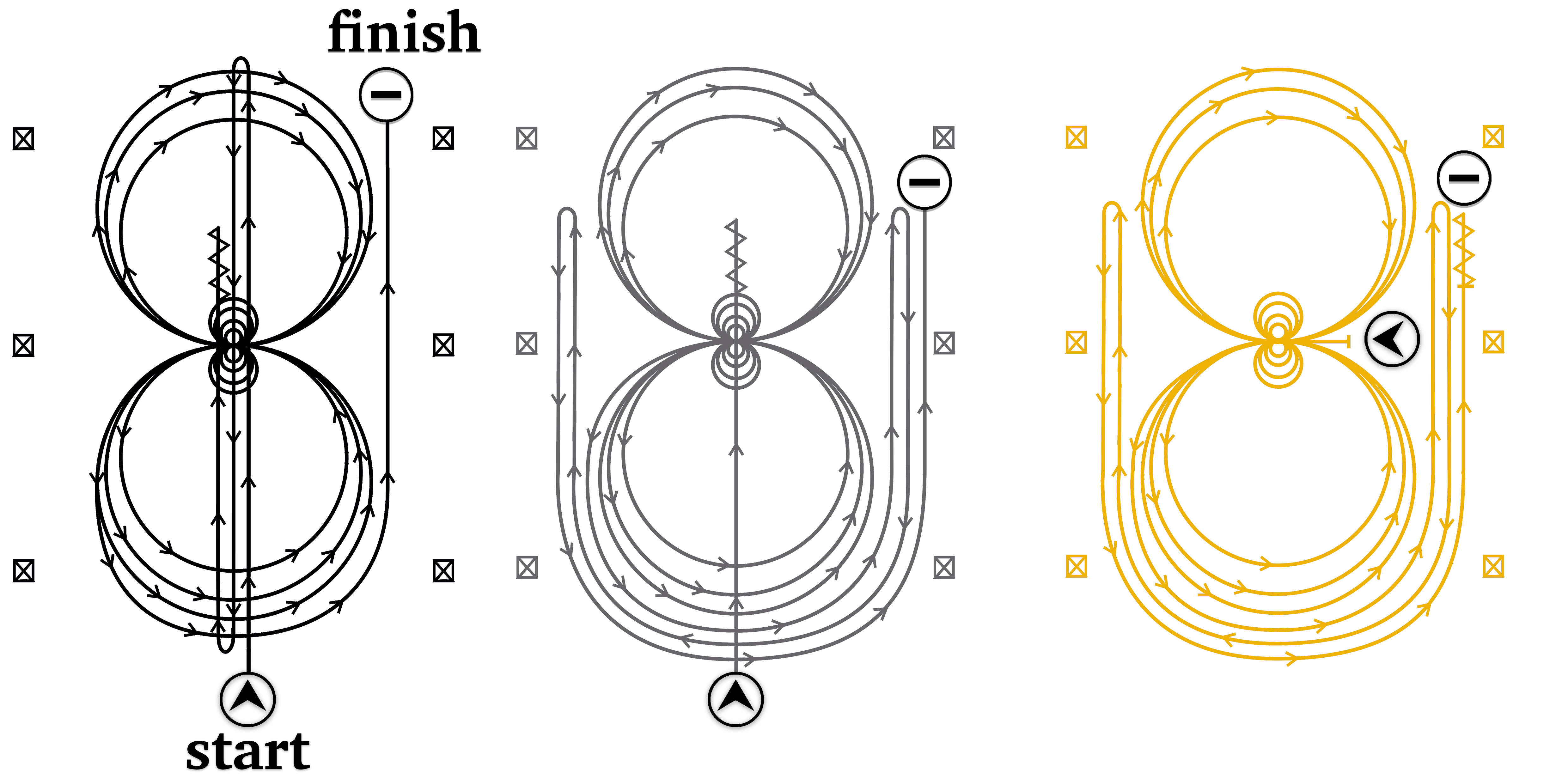

Supplement: Supplementary file 1 — Data S1: Schematic representation of three official reining patterns illustrating typical maneuver sequences performed in competition. Diagrams indicate the relative positioning of arena markers (center and side markers at ~15 m from end walls), directional arrows representing horse movement, and standardized elements such as spins, circles of varying speed, sliding stops, rollbacks, and lead changes. [file ASJ-97-e70223-s001.tif]
