# Supplementary material for: Phenotypic Definition Influences Genetic Inference and Predictive Ability for Reining Performance in Quarter Horses Using Random Regression Models
Source: Anim Sci J. 2026 Jul 23;97(1):e70223. doi: 10.1111/asj.70223 (PMC13392984; doi:10.1111/asj.70223)
Supplement: Supplementary file 2 — Data S2: Posterior summaries of variance components by age for within‐group rank (RANK) and Blom‐transformed rank (BLOM) of reining performance in Quarter Horses. Values are posterior means, standard deviations (SD), and 95% highest posterior density intervals (HPD95%) for rider, additive genetic, permanent environmental, and residual variance components obtained from a cubic random regression model. [file ASJ-97-e70223-s002.docx]

**Supporting Information S2**. Posterior summaries of variance components by age for within-group rank (RANK) and Blom-transformed rank (BLOM) of reining performance in Quarter Horses. Values are posterior means, standard deviations (SD), and 95% highest posterior density intervals (HPD95%) for rider, additive genetic, permanent environmental, and residual variance components obtained from a cubic random regression model.

| Age (years) | Rider | | | Additive genetic | | | Permanent environment | | | Residual | | |
| --- | --- | --- | --- | --- | --- | --- | --- | --- | --- | --- | --- | --- |
|  | Mean | SD | HPD95% | Mean | SD | HPD95% | Mean | SD | HPD95% | Mean | SD | HPD95% |
| RANK |  |  |  |  |  |  |  |  |  |  |  |  |
| 3 | 115.74 | 9.91 | 97.40 to 136.50 | 112.32 | 36.25 | 42.64 to 187.43 | 189.92 | 29.86 | 132.25 to 249.27 | 339.98 | 3.75 | 332.70 to 347.40 |
| 4 | 115.74 | 9.91 | 97.40 to 136.50 | 41.68 | 15.54 | 16.47 to 74.84 | 102.52 | 13.84 | 75.65 to 129.10 | 339.98 | 3.75 | 332.70 to 347.40 |
| 5 | 115.74 | 9.91 | 97.40 to 136.50 | 19.61 | 9.29 | 5.25 to 41.88 | 118.44 | 12.99 | 93.20 to 144.16 | 339.98 | 3.75 | 332.70 to 347.40 |
| 6 | 115.74 | 9.91 | 97.40 to 136.50 | 7.33 | 4.63 | 2.32 to 20.12 | 74.69 | 10.14 | 55.87 to 95.91 | 339.98 | 3.75 | 332.70 to 347.40 |
| 7 | 115.74 | 9.91 | 97.40 to 136.50 | 16.57 | 7.72 | 5.38 to 35.91 | 79.41 | 14.45 | 52.37 to 108.76 | 339.98 | 3.75 | 332.70 to 347.40 |
| 8 | 115.74 | 9.91 | 97.40 to 136.50 | 33.25 | 17.28 | 9.94 to 75.17 | 106.29 | 21.44 | 65.49 to 149.78 | 339.98 | 3.75 | 332.70 to 347.40 |
| 9 | 115.74 | 9.91 | 97.40 to 136.50 | 38.35 | 17.64 | 7.88 to 78.96 | 32.94 | 6.08 | 21.37 to 45.18 | 339.98 | 3.75 | 332.70 to 347.40 |
| 10 | 115.74 | 9.91 | 97.40 to 136.50 | 125.92 | 64.32 | 30.56 to 274.52 | 123.36 | 28.19 | 76.38 to 181.23 | 339.98 | 3.75 | 332.70 to 347.40 |
| BLOM |  |  |  |  |  |  |  |  |  |  |  |  |
| 3 | 27.68 | 2.30 | 23.47 to 32.43 | 10.57 | 2.98 | 3.81 to 16.13 | 22.82 | 2.82 | 17.64 to 28.84 | 48.60 | 0.54 | 47.56 to 49.66 |
| 4 | 27.68 | 2.30 | 23.47 to 32.43 | 10.88 | 3.34 | 5.19 to 18.11 | 19.6 | 2.71 | 14.13 to 24.69 | 48.60 | 0.54 | 47.56 to 49.66 |
| 5 | 27.68 | 2.30 | 23.47 to 32.43 | 10.95 | 3.22 | 5.68 to 18.14 | 22.82 | 2.93 | 16.95 to 28.48 | 48.60 | 0.54 | 47.56 to 49.66 |
| 6 | 27.68 | 2.30 | 23.47 to 32.43 | 11.33 | 4.03 | 5.36 to 21.54 | 18.04 | 3.56 | 9.97 to 24.74 | 48.60 | 0.54 | 47.56 to 49.66 |
| 7 | 27.68 | 2.30 | 23.47 to 32.43 | 13.42 | 6.32 | 4.78 to 30.59 | 25.69 | 6.16 | 10.34 to 36.51 | 48.60 | 0.54 | 47.56 to 49.66 |
| 8 | 27.68 | 2.30 | 23.47 to 32.43 | 10.91 | 6.38 | 3.00 to 27.58 | 34.81 | 7.78 | 16.63 to 48.93 | 48.60 | 0.54 | 47.56 to 49.66 |
| 9 | 27.68 | 2.30 | 23.47 to 32.43 | 6.50 | 4.43 | 1.10 to 18.10 | 27.6 | 5.55 | 18.04 to 39.95 | 48.60 | 0.54 | 47.56 to 49.66 |
| 10 | 27.68 | 2.30 | 23.47 to 32.43 | 53.70 | 19.5 | 17.30 to 104.67 | 94.8 | 16.13 | 62.66 to 193.54 | 48.60 | 0.54 | 47.56 to 49.66 |
